# Synthetic lethality guiding selection of drug combinations in ovarian cancer

**DOI:** 10.1371/journal.pone.0210859

**Published:** 2019-01-25

**Authors:** Andreas Heinzel, Maximilian Marhold, Paul Mayer, Michael Schwarz, Erwin Tomasich, Arno Lukas, Michael Krainer, Paul Perco

**Affiliations:** 1 Emergentec biodevelopment GmbH, Vienna, Austria; 2 Department of Internal Medicine III, Medical University of Vienna, Vienna, Austria; 3 Department of Oncology, Division of Internal Medicine I, Medical University of Vienna, Vienna, Austria; 4 Department of Internal Medicine IV, Medical University of Innsbruck, Innsbruck, Austria; University of South Alabama Mitchell Cancer Institute, UNITED STATES

## Abstract

**Background:**

Synthetic lethality describes a relationship between two genes where single loss of either gene does not trigger significant impact on cell viability, but simultaneous loss of both gene functions results in lethality. Targeting synthetic lethal interactions with drug combinations promises increased efficacy in tumor therapy.

**Materials and methods:**

We established a set of synthetic lethal interactions using publicly available data from yeast screens which were mapped to their respective human orthologs using information from orthology databases. This set of experimental synthetic lethal interactions was complemented by a set of predicted synthetic lethal interactions based on a set of protein meta-data like e.g. molecular pathway assignment. Based on the combined set, we evaluated drug combinations used in late stage clinical development (clinical phase III and IV trials) or already in clinical use for ovarian cancer with respect to their effect on synthetic lethal interactions. We furthermore identified a set of drug combinations currently not being tested in late stage ovarian cancer clinical trials that however have impact on synthetic lethal interactions thus being worth of further investigations regarding their therapeutic potential in ovarian cancer.

**Results:**

Twelve of the tested drug combinations addressed a synthetic lethal interaction with the anti-VEGF inhibitor bevacizumab in combination with paclitaxel being the most studied drug combination addressing the synthetic lethal pair between VEGFA and BCL2. The set of 84 predicted drug combinations for example holds the combination of the PARP inhibitor olaparib and paclitaxel, which showed efficacy in phase II clinical studies.

**Conclusion:**

A set of drug combinations currently not tested in late stage ovarian cancer clinical trials was identified having impact on synthetic lethal interactions thus being worth of further investigations regarding their therapeutic potential in ovarian cancer.

## Introduction

The concept of synthetic lethality (synlet) describes a relationship between two genes where single loss of either gene does not trigger significant impact on cell viability, but simultaneous loss of both gene functions results in lethality [1][2]. The term was introduced by Dobzhansky in 1946 studying mutations in fruit flies and identifying that specific combinations of mutations resulted in a lethal phenotype [3].

For cancer therapy, this principle can be utilized in different ways. One option is to address a tumor bearing a non-functional protein (either via mutation or downregulation) with a drug targeting a gene product being in a synthetic lethal interaction with the non-functional gene product [4]. Such approach promises improving safety in case the drug is not addressing a vital target mechanism. Another option is to use drug combinations where each drug is targeting a tumor-specific entity which in combination are synthetic lethal [5]. This latter approach promises increased efficacy and was also discussed in other disease areas like multiple sclerosis or lupus nephritis [6].

A prominent representative of using a synthetic lethal interaction in cancer therapy is the relationship between BRCA and PARP [7], [8]. Both are involved in DNA repair, with the BRCA pathway addressing repair of double-strand DNA breaks and PARP of single-strand DNA breaks A germline mutation in BRCA increases the risk for a variety of different cancers, but most notably breast and ovarian cancer, and accounts for more than 10% of all ovarian cancers [9]. In case of a second mutation or more likely chromosomal loss, the function of the BRCA pathway is lost and tumor develops. By pharmacologically targeting the synthetic lethal partner, PARP, the cytotoxic effect mainly occurs in tumor cells with hampered BRCA function, hence selectively eliminating tumor cells and exhibiting limited cytotoxic effect in non-cancerous tissue due to its still functional BRCA [10]. Only recently this concept has been proven ultimately successful in the clinic for the first time, with the FDA approval of the PARP inhibitor Olaparib as monotherapy in patients with deleterious germline BRCA-mutated advanced ovarian cancer treated with three or more lines of chemotherapy [11].

In the alternative scenario the synergy of two drugs is used for triggering a synthetic lethal interaction regardless of a specific mutation. According to the concept, if two drugs whose respective targets are in a synthetic lethal interaction are given at the same time the effect should be more than additive. Several such synergistic combinations are in clinical testing to date, e.g. the combination of Rucaparib and Temozolomide [12].

Research groups have started to exploit the concept of synthetic lethality in order to search for novel drug targets or drug combinations [4], [6].

We in this work systematically analyze drug combinations currently in clinical use or investigated in late stage phase III or IV clinical trials on ovarian cancer for a potential synthetic lethal background utilizing a consolidated set of synthetic lethal interactions derived from model organisms and from computational inference. Next to deciphering potential synthetic lethality in such given combinations we propose additional drug combinations potentially exhibiting a synthetic lethal mechanism of action for ovarian cancer.

## Materials and methods

### Cancer drug combinations in ovarian cancer

Drug combinations in ovarian cancer were extracted from clinical guidelines and late stage clinical trials. Clinical trial information was retrieved from ClinicalTrials.gov (https://clinicaltrials.gov/) using the search term “ovarian cancer” and limiting the search results to clinical trials in phase III and IV, i.e. for drug combinations where first evidence on efficacy from phase II trials was already available. Clinical trials holding one of the following terms in the condition field were further considered as relevant: “ovarian cancer”, “ovarian neoplasms”, “neoplasm of ovary”, “ovarian carcinoma”, “ovarian epithelial cancer”, “neoplasms, ovarian”. This step was necessary to exclude trials for example studying hypertension in a cohort of ovarian cancer patients. We further focused on clinical trials of type intervention with at least one drug combination consisting of drugs and/or biologicals. Drug names were mapped to their respective entries in DrugBank (version 4.3) where applicable [https://www.drugbank.ca/]. We in this manuscript from now on refer to this set of drugs and drug combinations as “tested drugs” and “tested drug combinations” respectively.

### Cancer drug targets

Drug target information was retrieved from DrugBank version 4.3 using the downloadable XML file as well as from the Therapeutic Target Database [http://bidd.nus.edu.sg/group/cjttd/] for those without target information in DrugBank using the TTD web search functionality. The list of drug and drug targets was further completed with drug targets identified as reported in literature for the set of drugs not represented in either of the two drug databases.

### Synthetic lethal interactions

Genetic interactions for Saccharomyces cerevisiae were retrieved from the supplementary website published by Costanzo et al. available at http://boonelab.ccbr.utoronto.ca/supplement/costanzo2009/ [13]. In particular the supplementary data file S1 was downloaded for identifying negative genetic interactions (i.e. synthetic lethal protein coding gene pairs) setting the cutoffs for p-value and genetic interaction scores to 0.05 and -0.08, respectively. Human orthologs of yeast genes were retrieved utilizing roundup [14], oma browser [15], ensembl [16], inparanoid [17] and HomoloGene [18]. Endpoints of yeast synthetic lethal interactions were mapped to their respective human genes, including them in further analysis only if at least one such orthologous gene could be identified. This procedure provided 204,124 synthetic lethal interactions between 3,714 human genes.

For further expanding the set of synthetic lethal interactions retrieved from yeast a computational inference method was applied. Mapping given synthetic lethal interactions on nodes (identifying protein coding genes) of a hybrid human protein coding gene interaction network [19] resulted in 194,565 interactions involving 3,530 network nodes. Given ortholog information from yeast allowing labelling of interaction pairs in the network enriched with node annotation including KEGG [20] and PANTHER pathway identifiers [21], Gene Ontology assignment according to PIR slim [22] (pruned to ancestor terms), disease association according to NCBI MeSH terms (http://www.ncbi.nlm.nih.gov/mesh), and drug association according to DrugBank (target, enzyme, transporter and carrier associations) was used to build a classifier allowing inference of putative synthetic lethal interactions. 186 gene nodes being member of synthetic lethal interactions met this high level of annotation (i.e. data from all mentioned sources was available). Next to annotation, topological properties were added for describing synthetic lethal interactions, including degree centrality, betweenness centrality, closeness centrality and local clustering coefficient, complemented by shortest paths. For learning a model on synthetic lethal interactions, Dice's coefficients for annotation of endpoints, the mean of node-based graph-statistics and the shortest path between synthetic lethal nodes were used. Non-synthetic lethal interactions were sampled at random between highly-annotated end points not taking part in any known synthetic lethal interaction, thereby achieving a balanced set of instances holding 1,049 synthetic lethal-positive and an equivalent number of synthetic lethal-negative cases. This set was split into a balanced training set (2/3 of all instances) and a test set (1/3 of all instances). Using WEKA [http://www.cs.waikato.ac.nz/ml/index.html], a Random Forest model [23] was derived on the training set (performing parameter-optimization using 10-fold cross-validation) and validated on the test set. The final model consisting of 93 trees achieved 76.5% accuracy on the test set (with a precision of 0.77, a recall of 0.757, a F1-score of 0.774, a specificity of 0.860 and an AUC of 0.757). With this procedure 135,400 synthetic lethal interactions between 1,060 human genes could be predicted. Combining the synthetic lethal interaction data set derived from yeast ortholog mapping and computational inference resulted in 4,556 human genes being involved in 339,524 synthetic lethal interactions.

### Evaluation of drug combinations and proposal of new ones

We searched for tested drug combinations addressing a synlet interaction, i.e. synlet interactions were selected where both synlet interactors are direct drug targets of tested drugs being combined in one of the tested drug combinations. Focus was on synlet pairs where both synlet interactors are addressed by two different tested drugs with no single drug available targeting both synlet interactors. All pairwise drug combinations were constructed for evaluation for tested drug combinations holding more than two drugs.

For proposing novel drug combinations targeting synlet interactions we were looking for exactly such kind of synlet pairs using the set of individual drugs from the set of tested drugs.

The annotation degree of the respective synlet interaction partners for ovarian cancer was used in order to rank proposed synlet interactions. Annotation degree was defined as the sum of resulting publications from a PubMed search for the respective proteins using the gene symbol as well as the gene name in combination with the major MeSH term ovarian neoplasms.

## Results and discussion

### Cancer drug combinations and addressed drug targets

**Fig 1:**
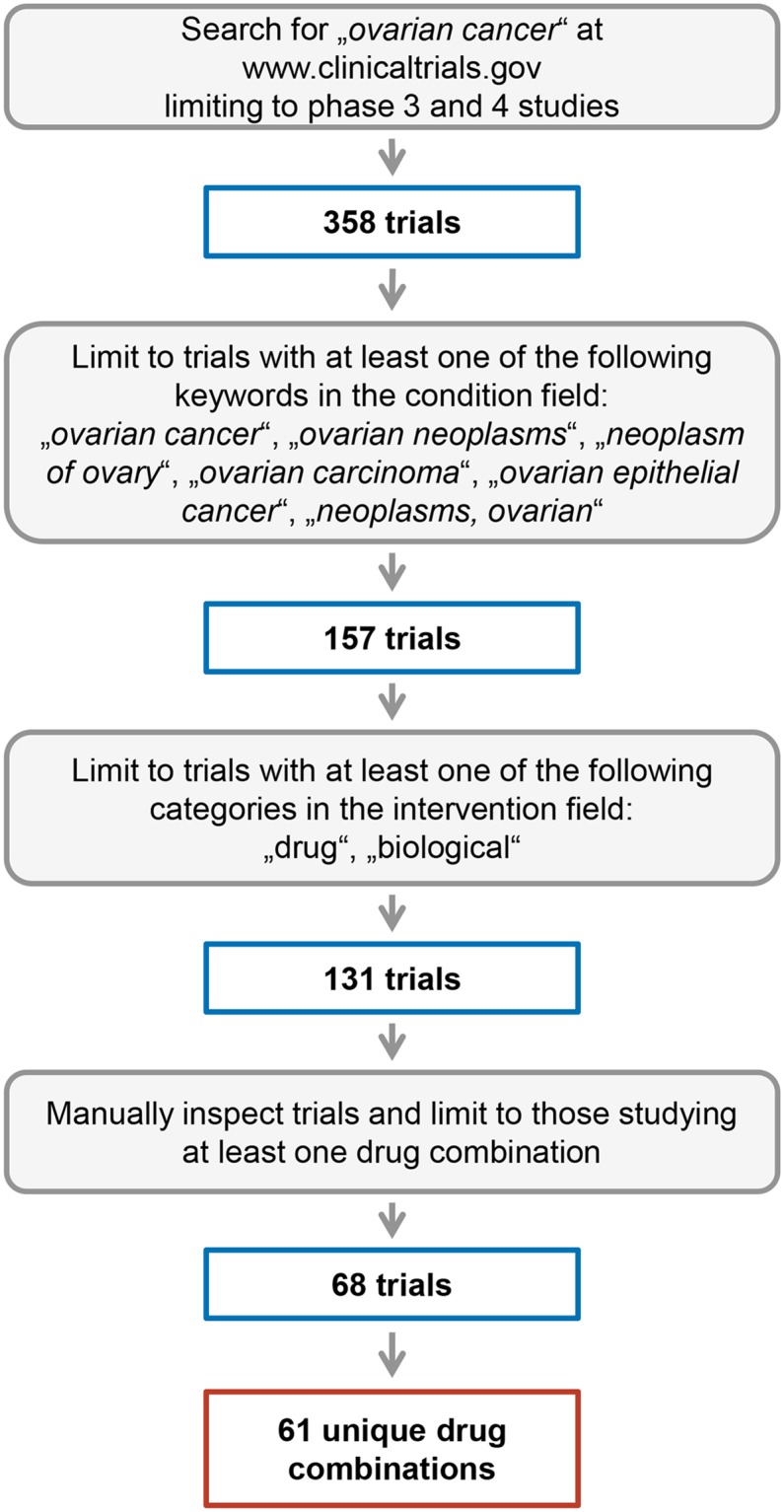
Ovarian cancer clinical trial workflow. Fig 1 depicts an overview on the search strategy to identify relevant ovarian cancer clinical trials and drug combinations along with numbers of the individual processing steps.

We in total identified 68 phase III or IV clinical trials on ovarian cancer studying at least one combination of drugs/biologicals. 95 drug combinations were studied in total in these 68 trials with 61 unique drug combinations. A schematic representation of the workflow for processing clinical trial data is given in Fig 1.

**Fig 2:**
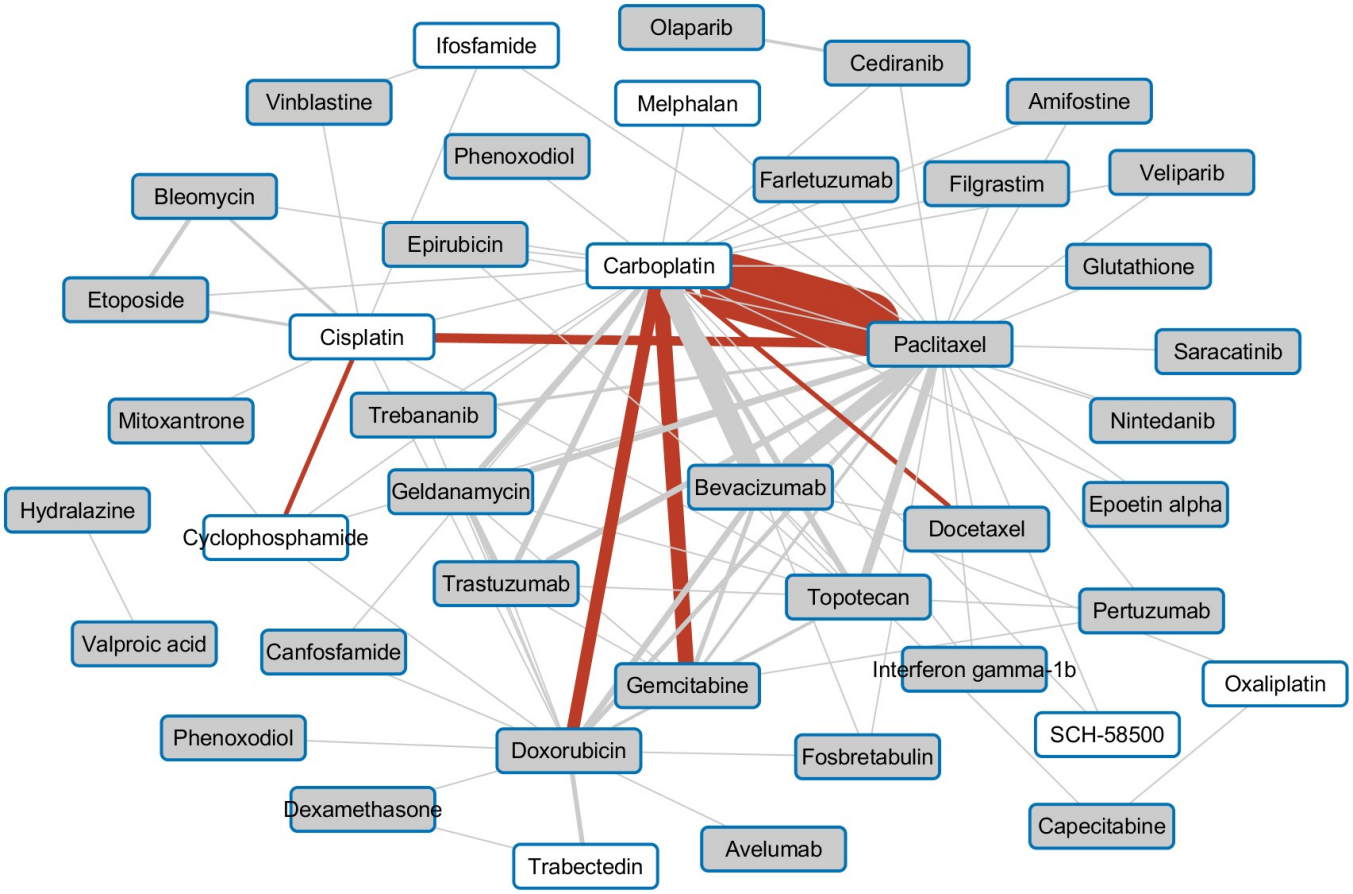
Drug-drug combination map. Fig 2 depicts the pairwise drug-drug combinations extracted from phase III and IV ovarian cancer clinical trials of the 43 drugs used in these combinations. The edge width corresponds to the number of the studied drug-drug combination with combinations already used in the clinical setting indicated by a red edge. Drugs having at least one protein target assigned have a grey background, whereas drugs not directly targeting specific proteins but merely interfering with DNA are displayed with a white background.

The most widely investigated drug combination, tested in 39 clinical trials, consisted of carboplatin, a platinum-based drug, and paclitaxel, the current first line therapy in high-grade serous ovarian cancer [24]. Other combinations already used in clinical practice being tested in a number of clinical studies center around chemotherapeutics like the combination of carboplatin and gemcitabine or the combination of carboplatin and doxorubicin. From the set of targeted agents the vascular endothelial growth factor A (VEGFA) inhibitor bevacizumab stands out being investigated primarily in combination with carboplatin and/or paclitaxel [25], [26]. The map of pairwise drug combinations being investigated in late stage clinical trials is depicted in Fig 2.

35 of the 43 drugs used in the set of tested drug combinations had at least one protein target with an average number of 4.5 protein targets per drug. The drug with the largest number of protein targets was Glutathione with 37 drug targets assigned followed by valproic acid with a set of 20 drug targets. Glutathione however rather serves as substrate than as inhibitor for most of the listed drug targets. Chemotherapeutic agents like e.g. cisplatin, carboplatin, or melphalan on the other hand having solely DNA as target with no direct protein targets available (drugs with white background in Fig 2) had to be excluded from further analysis as the synlet interactions are always composed of two protein coding genes. Analysis with respect to addressing synlet interactions was thus focused on the set of 35 drugs having at least one protein drug target.

### Given drug combinations addressing synthetic lethal pairs

**Fig 3:**
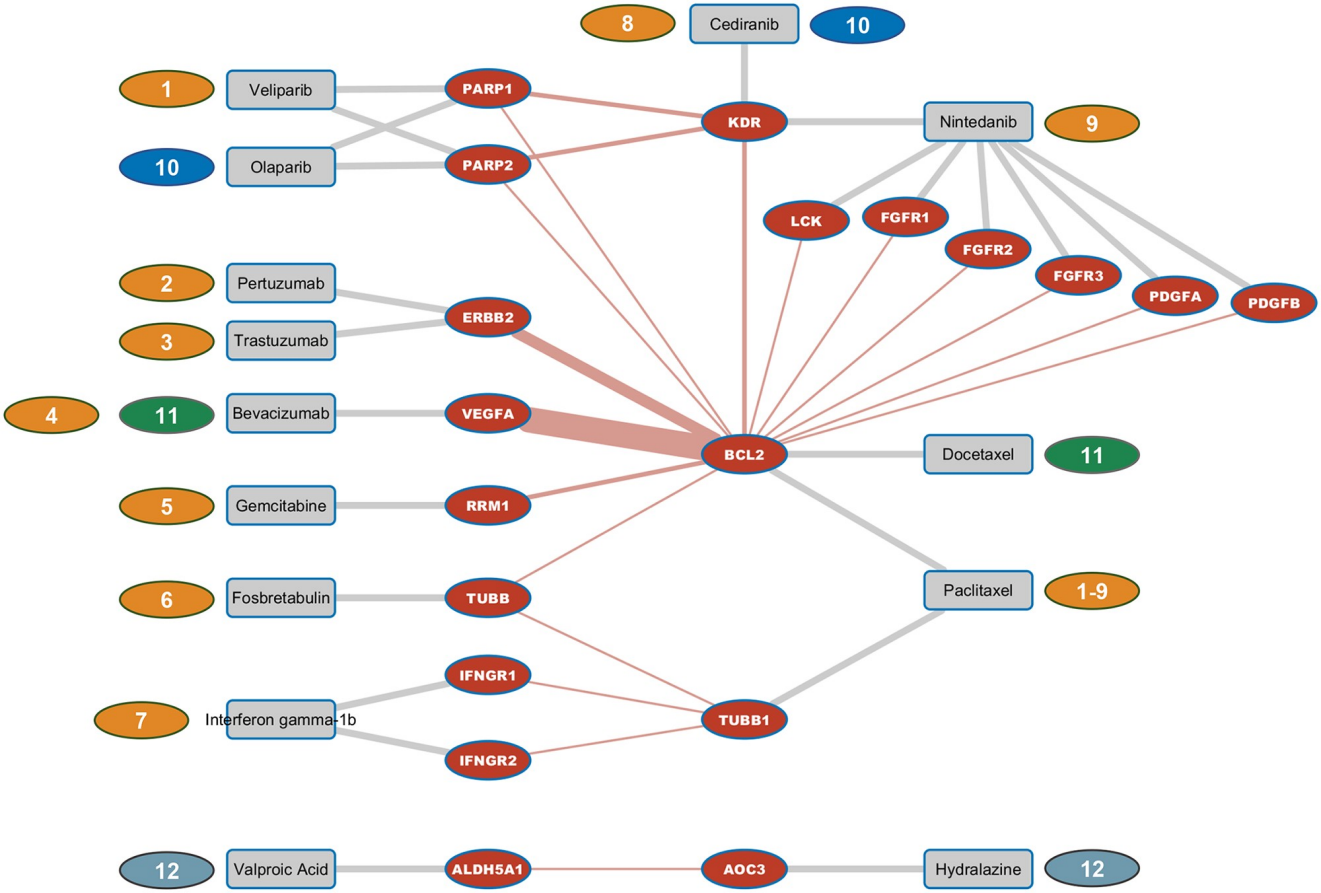
Synlet map of known drug combinations. Fig 3 depicts synlet pairs addressed by drug combinations either already approved or in late stage clinical testing. Color-coded number tags indicate the twelve pairwise drug combinations. Synlet edges are displayed in red with drug target links shown in grey color. The width of synlet edges corresponds to the number of trials available testing drug combinations addressing the respective synlet interaction.

Twelve of the tested drug combinations addressed a synlet interaction as depicted in Fig 3. Nine of the twelve tested drug combinations involved paclitaxel in combination with either veliparib, pertuzumab, trastuzumab, bevacizumab, gemcitabine, fosbretabulin, interferone gamma-1b, cediranib, or nintedanib respectively. Bevacizumab in addition to the combination with paclitaxel also addressed the synlet pair VEGFA and BCL2 in combination with docetaxel. The PARP inhibitor olaparib in combination with cediranib addressed the synlet pairs PARP1 and KDR as well as PARP2 and KDR. Drug combination number twelve consisted of valproic acid and hydralazine, targeting the synlet pair ALDH5A1 and AOC3.

As mentioned before, the widely used platinum-based therapies with cisplatin, carboplatin or oxaliplatin were not considered in this analysis because of unspecific cross-linking guanine bases in DNA thus impairing DNA replication with no direct protein drug targets available. Of the twelve synlet interactions addressed by the tested drug combinations BCL2 was involved in ten being addressed by either paclitaxel or docetaxel. Although not being the main target of paclitaxel, BCL2 has been shown to be negatively affected by paclitaxel in tumor cell lines but also in regulatory T cells [27], [28]. The synlet interaction between BCL2 and VEGFA is of particular interest as the combination of paclitaxel and bevacizumab is a heavily tested one. VEGFA was previously proposed as predictive marker for mTOR inhibition in the context of high-grade serous ovarian cancer therapy [29]. Based on results from the phase III ICON7 study, bevacizumab had no effect on overall survival in the whole study population of newly diagnosed ovarian cancer patients but had a positive effect on overall survival in poor-prognosis patients [25]. This is another example where a drug’s efficacy only affects a subgroup of patients thus highlighting the need for molecular markers being capable of stratifying patients with respect to drug response.

The PARP inhibitor veliparib in combination with paclitaxel was found to address the synlet interactions between PARP1 and PARP2 with BCL2 respectively. An interaction on the molecular level between BCL2 and PARP1 in tumor cell lines was shown by Dutta and colleagues thus making this interaction an interesting therapeutic target in cancer [30]. A molecular link between BCL2 and ERBB2 was also found in breast cancer cell lines making also this synlet interaction interesting in the context of ovarian cancer treatment [31] with tested drug combinations addressing this synlet pair being trastuzumab and paclitaxel as well as pertuzumab and pacliataxel.

A clinical benefit could be shown for the combination of the epigenetic agents valproic acid and hydralazine in combination to chemotherapy when tested in a small cohort of tumor patients by Candelaria and colleagues [32].

Despite the fact that only a small number of tested pairwise drug combinations affect synlet interactions, these combinations might be of particular interest when assessing the status of the synlet target genes thus potentially identifying those patients benefitting the most from these combinations.

### Novel proposed drug combinations addressing synthetic lethal pairs

**Fig 4:**
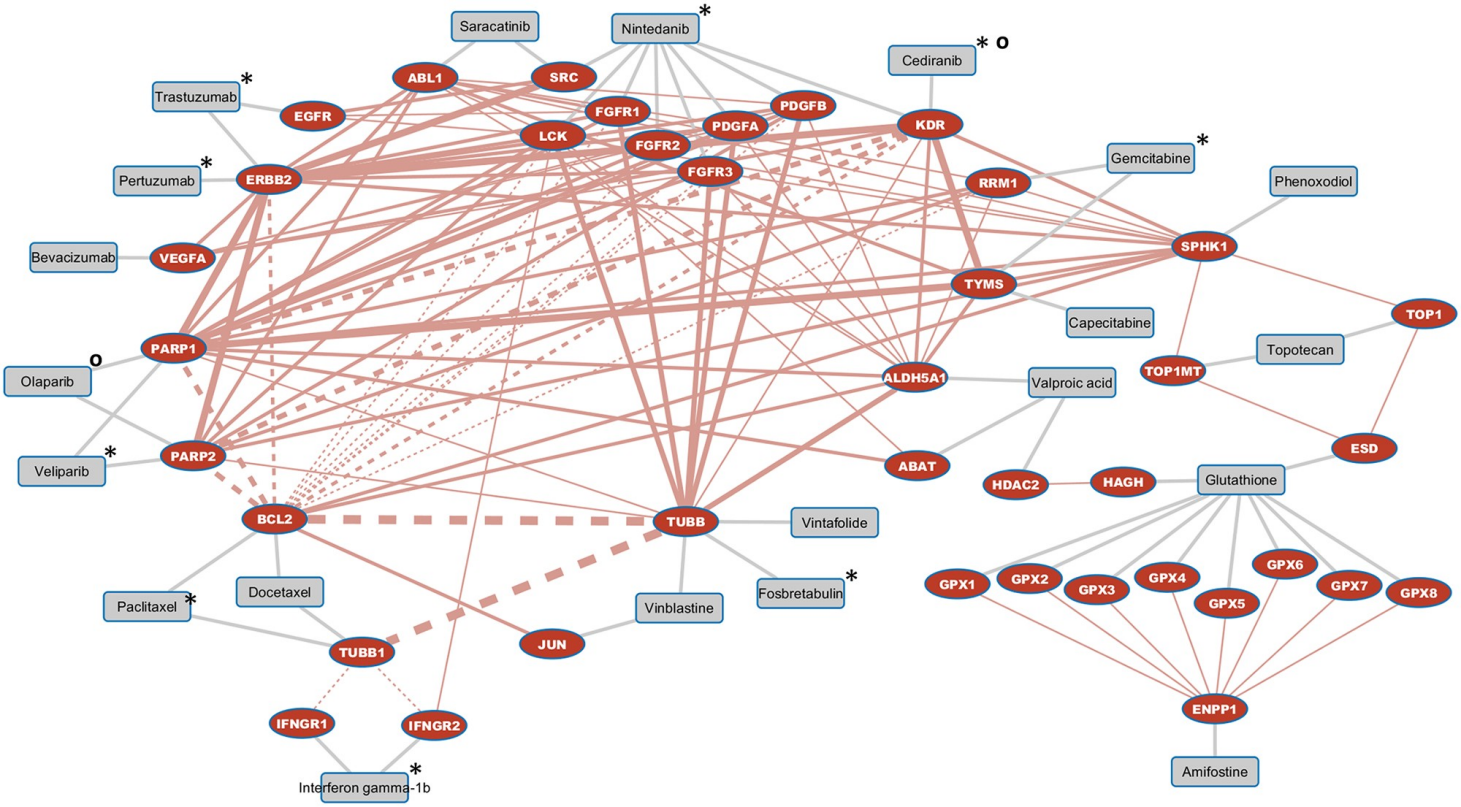
Synlet map of novel proposed drug combinations. Fig 4. depicts synlet pairs being eligible to be addressed by drug combinations currently not in clinical use or late stage clinical testing. Each two drugs targeting the two synlet partners can be considered as drug combination. Synlet edges are displayed in red with drug target links shown in grey color. The width of synlet edges corresponds to the number of drug combinations targeting this specific synlet interaction. Synlet interactions already addressed by drug combinations in clinical use or late stage testing are indicated by dotted lines. These drug combinations are composed of those drugs flagged with an asterisk (*) each in combination with paclitaxel always via the path through the dotted synlet interaction or with a circle (**o**) for the combination between cediranib and olaparib.

We identified a set of 84 drug combinations currently not tested in phase III or IV trials in the context of ovarian cancer which address synlet interactions and are thus of interest to be further evaluated for the treatment of ovarian cancer. 102 synlet interactions in total are addressed by the 84 drug combinations, with 17 synlet interactions already addressed by tested drug combinations and the remaining 85 synlet interactions not addressed so far by tested drug combinations as depicted in Fig 4.

The following synlet interactions are addressable by six drug combinations each, namely PARP1 with TUBB, PARP2 with TUBB, as well as KDR with TUBB. Synlet interactions addressed by five novel drug combinations are BCL2 with TUBB as well as TUBB with TUBB1. The synlet pairs PARP1 with BCL2 and PARP2 with BCL2 are addressable by three novel drug combinations in addition to the already tested combination of paclitaxel and veliparib which we discussed in the chapter on tested drug combinations. Other synlet interactions already addressed by tested drug combinations with other novel drug combinations available are among others BCL2 with ERBB2 and RRM1 as well as TUBB1 with IFNGR1 and IFNGR2. These synlet interactions are depicted with dotted lines in Fig 4 as drug combinations addressing these synlet interactions do not depict novel mechanisms.

All novel drug combinations targeting synlet interactions are listed in the S1 Table sorted in descending order based on the sum of publications retrieved for papers annotation with the major MeSH term ovarian neoplasms for both synlet interaction partners with the top ranked drug combinations given in Table 1.

**Table 1:** Synlet interactions addressed by novel drug combinations. The top addressed synlet interactions based on sum of PubMed ID (PMID) counts as well as the respective novel proposed drug combinations are given in Table 1. Synlet interactions are sorted in descending order based on the sum of PMID counts of the two synlet interacting partners in the context of ovarian cancer. PMID counts of the two synlet interaction partners are also provided in brackets for synlet partner A and B respectively.

Drug A	Drug B	Synlet partner A	Synlet partner B	PMID counts
Pertuzumab	Bevacizumab	ERBB2	VEGFA	1262 (695 / 567)
Trastuzumab	Bevacizumab	ERBB2	VEGFA	1262 (695 / 567)
Pertuzumab	Saracatinib	ERBB2	SRC	808 (695 / 113)
Pertuzumab	Nintedanib	ERBB2	SRC	808 (695 / 113)
Trastuzumab	Nintedanib	ERBB2	SRC	808 (695 / 113)
Trastuzumab	Saracatinib	ERBB2	SRC	808 (695 / 113)
Pertuzumab	Docetaxel	ERBB2	BCL2	761 (695 / 66)
Trastuzumab	Docetaxel	ERBB2	BCL2	761 (695 / 66)
Trastuzumab	Nintedanib	EGFR	SRC	749 (636 / 113)
Trastuzumab	Saracatinib	EGFR	SRC	749 (636 / 113)
Pertuzumab	Olaparib	ERBB2	PARP1	736 (695 / 41)
Pertuzumab	Veliparib	ERBB2	PARP1	736 (695 / 41)
Trastuzumab	Veliparib	ERBB2	PARP1	736 (695 / 41)
Trastuzumab	Olaparib	ERBB2	PARP1	736 (695 / 41)
Pertuzumab	Nintedanib	ERBB2	FGFR2	719 (695 / 24)
Trastuzumab	Nintedanib	ERBB2	FGFR2	719 (695 / 24)
Pertuzumab	Nintedanib	ERBB2	PDGFB	718 (695 / 23)
Trastuzumab	Nintedanib	ERBB2	PDGFB	718 (695 / 23)
Pertuzumab	Cediranib	ERBB2	KDR	717 (695 / 22)
Pertuzumab	Nintedanib	ERBB2	KDR	717 (695 / 22)
Trastuzumab	Cediranib	ERBB2	KDR	717 (695 / 22)
Trastuzumab	Nintedanib	ERBB2	KDR	717 (695 / 22)
Pertuzumab	Nintedanib	ERBB2	PDGFA	714 (695 / 19)
Trastuzumab	Nintedanib	ERBB2	PDGFA	714 (695 / 19)
Pertuzumab	Nintedanib	ERBB2	FGFR1	713 (695 / 18)
Trastuzumab	Nintedanib	ERBB2	FGFR1	713 (695 / 18)
Pertuzumab	Saracatinib	ERBB2	ABL1	705 (695 / 10)
Trastuzumab	Saracatinib	ERBB2	ABL1	705 (695 / 10)
Pertuzumab	Nintedanib	ERBB2	FGFR3	702 (695 / 7)
Trastuzumab	Nintedanib	ERBB2	FGFR3	702 (695 / 7)
Pertuzumab	Nintedanib	ERBB2	LCK	702 (695 / 7)
Trastuzumab	Nintedanib	ERBB2	LCK	702 (695 / 7)
Pertuzumab	Olaparib	ERBB2	PARP2	697 (695 / 2)
Pertuzumab	Veliparib	ERBB2	PARP2	697 (695 / 2)
Trastuzumab	Veliparib	ERBB2	PARP2	697 (695 / 2)
Trastuzumab	Olaparib	ERBB2	PARP2	697 (695 / 2)
Pertuzumab	Phenoxodiol	ERBB2	SPHK1	697 (695 / 2)
Trastuzumab	Phenoxodiol	ERBB2	SPHK1	697 (695 / 2)
Trastuzumab	Nintedanib	EGFR	FGFR1	654 (636 / 18)
Trastuzumab	Nintedanib	EGFR	LCK	643 (636 / 7)
Vinblastine	Paclitaxel	JUN	BCL2	602 (536 / 66)
Vinblastine	Docetaxel	JUN	BCL2	602 (536 / 66)
Bevacizumab	Nintedanib	VEGFA	PDGFB	590 (567 / 23)
Bevacizumab	Nintedanib	VEGFA	KDR	589 (567 / 22)
Bevacizumab	Cediranib	VEGFA	KDR	589 (567 / 22)
Bevacizumab	Nintedanib	VEGFA	PDGFA	586 (567 / 19)
Bevacizumab	Nintedanib	VEGFA	FGFR3	574 (567 / 7)
Topotecan	Glutathione	TOP1	ESD	130 (128 / 2)
Topotecan	Phenoxodiol	TOP1	SPHK1	130 (128 / 2)
Docetaxel	Veliparib	BCL2	PARP1	107 (66 / 41)
Paclitaxel	Olaparib	BCL2	PARP1	107 (66 / 41)
Docetaxel	Olaparib	BCL2	PARP1	107 (66 / 41)

The top ranked combinations consisted of the anti-VEGF inhibitor bevacizumab together with one of the two ERBB2 inhibitors, namely trastuzumab or pertuzumab. This combination has already been tested in phase 2 clinical trials in the context of breast neoplasms along with the chemotherapeutic docetaxel among others [33], [34]. Docetaxel in combination with one of the ERBB2 inhibitors showed up very high in the ranking based on PubMed counts of the addressed synlet interaction partners.

Due to the fact that ERBB2 alone was reported in 697 publications on ovarian neoplasms also the synlet interactions to PARP1, PARP2 as well as SPHK1 were ranked very high in the list, addressed by the ERBB2 inhibitors with either one of the PARP inhibitors olaparib or veliparib or with phenoxodiol, the synthetic analog of genistein, targeting the sphingosine kinase 1 (SPHK1). SPHK1 was recently reported to be a therapeutic target of interest in high grade serous ovarian cancer as could be demonstrated by knock-out experiments in mice [35]. Phenoxodiol in combination with carboplatin however did not show evidence for clinical activity based on data of a phase III study in acquired platinum-resistant ovarian cancer [36].

The PARP inhibitor olaparib in combination with paclitaxel and carboplatin on the other hand was shown to significantly improve progression-free survival in a phase II clinical study [37]. The combination of olaparib and paclitaxel addresses the synlet interaction of PARP1 and BCL2, also ranked in the top third of synlet interactions in our analysis based on PMID counts of the two synlet partners.

The tyrosine-kinase inhibitor nintedanib is the most frequent drug in the list of novel drug combinations due to the fact of the multitude of drug targets addressed by the compound. Nintedanib is currently being investigated in a phase III trial in combination with paclitaxel and carboplatin for first line treatment of ovarian cancer [https://clinicaltrials.gov/show/NCT01015118]. Nintedanib is approved for idiopathic pulmonary fibrosis as well as non-small cell lung cancer and is tested in a number of trials on various cancer types [38], [39].

One of the more interesting combinations targeting not so well annotated protein in the context of ovarian cancer is the one between valproic acid in combination with either gemcitabine or vinblastine, targeting the synlet interactions of ALDH5A1 with RRM1 and TUBB respectively. Valproic acid was shown to enhance the cytotoxic effect on gynecologic cancer cells in combination with an aurora kinase inhibitor [40]. Valproic acid was also reported to sensitize gemcitabine-induced cytotoxicity in gemcitabine-resistant pancreatic cancer cells [41].

A limitation of the present study is the lack of experimental validation on identified novel drug combinations as the focus of this study was on the data integration pipeline with an application on established drug combinations in the context of ovarian cancer. In addition, the way we currently rank novel identified drug combinations based on the sum of available literature information for the two synthetic lethal gene partners is probably a little biased towards genes and proteins which in general are better annotated and characterized in scientific literature. A way to counterbalance this potential bias would be to calculate an enrichment score for each gene based on papers in the context of ovarian cancer as compared to the background set of publications for each gene. Another option would be to use measures such as the Gene Characterization Index to account for the different degrees of gene annotation [42]. Despite the fact that we used rather stringent criteria to define our synthetic lethal interaction set, one has to admit that synthetic lethal interactions were experimentally determined in yeast and not in human tissue. Research groups have in the meantime started to generate sets of synthetic lethal interactions in human tissues [43]. As described in a previous publication of our group [44], we used a random forest classification algorithm to extend the set of experimental synthetic lethal interactions to also include human genes not being present in the yeast genome.

Drug combinations addressing synthetic lethal interactions proposed in this study pave the way for further studies evaluating these combinations in more detail in order to enhance drug efficacy. Lee and colleagues demonstrated how such systems-based attacks with drug combinations can significantly increase drug efficacy, especially when administered in the proper order and timing of administration [45].

Further, preclinical proof-of-concept studies as published by our group for breast cancer [46] and previously performed in the field of ovarian cancer using patient-derived xenograft (PDX) or in-vitro models [47], [48], are dearly needed to add biological significance to the drug combinations predicted through our in-silico approach.

## Conclusions

In summary we on the one hand could show that drug combinations currently in late stage clinical testing address synthetic lethal interactions and on the other hand we identified a set of drug combinations currently not tested in late stage ovarian cancer clinical trials worth of further investigation due to their impact on synthetic lethal interactions.

## Supporting information

S1 Table Complete list of synlet interactions addressed by novel drug combinations. The full list of addressed synlet interactions as well as the respective novel proposed drug combinations are given in the S1 Table. Synlet interactions are sorted in descending order based on the sum of PubMed ID (PMID) counts of the two synlet interacting partners in the context of ovarian cancer. PMID counts of the two synlet interaction partners are also provided in brackets for synlet partner A and B respectively.
(XLSX)
